# Draft Genome Sequence of a Highly Flagellated, Fast-Swimming Archaeon, *Methanocaldococcus villosus* Strain KIN24-T80 (DSM 22612)

**DOI:** 10.1128/genomeA.00481-13

**Published:** 2013-07-11

**Authors:** Sugumar Thennarasu, Dineshreddy Polireddy, Aju Antony, Madhava Rao Yada, Sami Algarawi, Neelamegam Sivakumar

**Affiliations:** Bioscience Core Laboratory, King Abdullah University of Science and Technology (KAUST), Thuwal, Saudi Arabia

## Abstract

We report the draft genome sequence of a hyperthermophilic *Methanocaldococcus villosus* strain, KIN24-T80. The gene associated with its heavy flagellum formation was annotated in the 1.2-Mb draft genome sequence, and this strain may be a good model system to study the extensive functional role of flagella and their fast motor activity.

## GENOME ANNOUNCEMENT

*Methanocaldococcus villosus* strain KIN24-T80 is a chemolithoautotrophic, hyperthermophilic, Gram-negative anaerobic methanogen (1). It can grow between 55 and 90°C, with an optimum at 80°C. Cell shapes are regular to irregular cocci, 1 to 2 µm in diameter (1). *M. villosus* is a heavily flagellated archaeon that adheres to abiotic surfaces and forms cell-to-cell contacts (1). The archaeal flagellum has a unique motility apparatus distinct from the bacterial flagellum. *M. villosus* is currently found to be one of the fastest moving organisms reported in terms of speed measured in bodies per second (bps). Its swimming speed was measured close to 500 bps; this is much higher than the speed of *Escherichia coli* or the cheetah, each with a speed of 20 bps (2).

The genome of *M. villosus* strain KIN24-T80 was sequenced using semiconductor sequencing technology (Ion personal genome machine; Life Technologies). The sequence from the shotgun library was analyzed and assembled using CLC genomic workbench software. A total of 5,409,659 reads were subjected to quality control, and approximately 560 Mb of raw data with an average read length of 103 bp and with an average Phred score of 27 was selected for assembly. The sequences were assembled into 93 contigs with lengths ranging from 404 to 70,884 bases, giving a total genome size of 1.214 Mb. A total of 81 contigs with sizes of >700 bases were selected for genome submission. The G+C content of the genomic DNA was 29.40%.

The functional annotation was initially performed with the NCBI Prokaryotic Genomes Automatic Annotation Pipeline (PGAAP), along with the genome submission to NCBI. The sequences were also subjected to RAST (3) annotation, and it identified 1,422 coding sequences, of which 180 are uncharacterized and 203 encode hypothetical proteins. *M. villosus* strain KIN24-T80 is reported to have a large number of flagella, and these cell appendages mediate very fast motility, adherence to abiotic surfaces, and the formation of cell-cell contacts (1, 2). The gene cluster responsible for the large number of flagella was identified; the cluster contains 11 flagellum-related genes. The genes are arranged in the following order in the cluster, *flaB1-flaB2-flaB3-flaC-flaD-flaE-flaF-flaG-flaH-flaI-flaJ*, and this order is similar to that reported for the genetic locus of *Methanococcus maripaludis* (4). Comparative genome analysis performed using the RAST server revealed its closest neighbors as *Methanocaldococcus jannaschii* strain DSM 2661, *Methanococcus maripaludis* strain C7, and *Methanococcus vannielii* strain SB. Further genome analysis of this organism will help uncover biosynthetic pathways and enzymes that perform unique and valuable biotransformations.

### Nucleotide sequence accession numbers.

The annotated draft genome sequence was deposited in DDBJ/EMBL/GenBank under accession no. APMM00000000. The version described in this paper is the first version, APMM00000000.1.
